# Accident vasculaire cérébral ischémique révélant un syndrome de Fahr

**DOI:** 10.11604/pamj.2018.30.259.10787

**Published:** 2018-08-07

**Authors:** Nawfal Doghmi, Abdelghafour Elkoundi, Amine Belghiti, Abdelouahed Baite, Charki Haimeur

**Affiliations:** 1 Service de Réanimation Médicale, Pôle Anesthésie-Réanimation, Hôpital Militaire Mohamed V, Rabat, Maroc

**Keywords:** Syndrome de Fahr, épilepsie, accident vasculaire cérébrale ischémique, métabolisme phosphocalcique, Fahr’s syndrome, epilepsy, ischemic stroke, phosphocalcium metabolism

## Abstract

Le syndrome de Fahr est défini par la présence de calcifications intracérébrales bilatérales et symétriques, non artériosclérotiques, localisées aux noyaux gris centraux. La découverte de celles-ci au cours d’un accident cérébrale ischémique constitue un mode de révélation exceptionnelle de cette affection et doit faire pratiquer une étude du métabolisme phosphocalcique avec dosage de la PTH. L’analyse des signes clinico-biologiques et radiologique est à la base du diagnostic. Un traitement antiépileptique au long cours peut favoriser les calcifications au niveau des noyaux gris centraux et induire le syndrome qui reste de bon pronostic. La correction des troubles du métabolisme phosphocalcique amène souvent une amélioration notable.

## Abstract (en)

Fahr’s syndrome is defined as the presence of bilateral intracerebral, symmetrical, nonarteriosclerotic calcifications involving the basal ganglia. Their detection during stroke is exceptional. Phosphocalcium metabolism should be investigated with human parathyroid hormone (HPH) dosage. Diagnosis is based on the evaluation of clinical and laboratory data as well as on radiological imaging. Long-term antiepileptic treatment can cause basal ganglia calcification resulting in Fahr’s syndrome. Prognosis is favorable. The correction of phosphocalcium metabolism usually leads to significant improvement.

## Introduction

Le syndrome de Fahr a été défini par Theodor Fahr en 1930 par la présence de calcifications intracérébrales bilatérales et symétriques, non artériosclérotiques, localisées aux noyaux gris centraux [1]. Cette entité, dont la physiopathologie reste non élucidée, est associée à des troubles du métabolisme phosphocalcique et principalement à une hypoparathyroidie. Les accidents vasculaires cérébraux ischémiques (AVCI) n’ont jamais été décrits comme des signes révélateurs. Nous rapportons l’observation de syndrome de Fahr découvert suite à un AVCI et nous essayons d’établir une relation entre la pathologie vasculaire et cette entité rare avec revue de la littérature.

## Patient et observation

Il s’agissait d’un patient âgé de 51 ans, admis au service de réanimation pour un coma post critique. Dans ses antécédents on note une hypertension artérielle sous nicardipine, un diabète type 2 sous antidiabétiques oraux et un suivi pour épilepsie sous valproate de sodium depuis 10 ans. A noter qu'il n'y a pas de cas similaires dans la famille. 15 jours avant son admission, il présenta une dysarthrie associée à une difficulté de marcher et un déséquilibre permanent obligeant le malade à déambuler avec l’aide de béquilles. Le patient rapporta également des céphalées modérées ainsi que des troubles mnésiques quasi constants. 24h avant son admission à l’hôpital, le patient a présenté une paralysie faciale et une hémiparésie droite le motivant à se présenter à l’hôpital.

**Figure 1:**
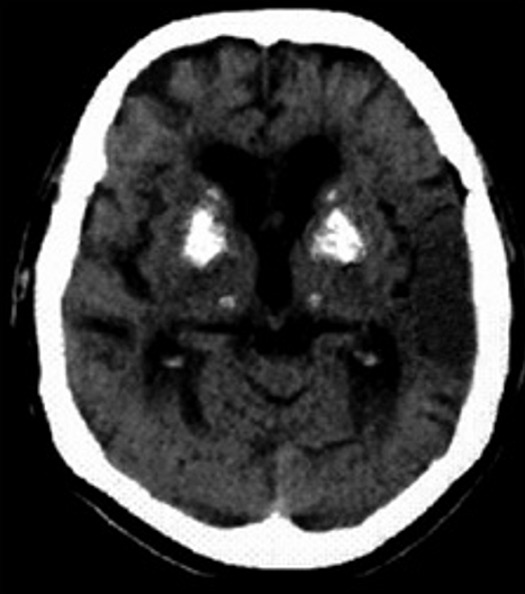
Calcifications bilatérale des noyaux gris centraux avec une hypodensité temporale gauche

A son admission, le patient était confus avec une hémiparésie du côté droit et une paralysie faciale constante. Les pupilles étaient de taille intermédiaire et réactives à la lumière. La nuque était souple et les réflexes ostéotendineux étaient vifs et symétriques. Le signe de Chvosteck et la manœuvre de trousseau étaient positifs. Par ailleurs, la pression artérielle était à 190/100mmHg, le pouls à 120 battements/minute, la fréquence respiratoire à 22 cycles/minute et la température à 37°C. Le reste de l’examen somatique était sans particularité. Le scanner cérébral sans injection (Figure 1) a révélé une atrophie cortico sous corticale avec une discrète hypodensité temporale gauche et des calcifications bilatérales des noyaux gris centraux.

Le diagnostic de maladie de Fahr a été évoqué et nous avons complété le bilan par un bilan phosphocalcique qui a révélé une hypocalcémie sévère à 1,2mmol.L-1 (valeur normale 2,2 et 2,6mmol·L-1), une phosphorémie à 1,40mmol/L (valeur normal 0.80 - 1.45mmol·L-1). Un dosage de la vit 25 OH D2 était à 17ng/ml (valeur normale 30-100 ng/ml), alors que le dosage de la PTH était normal éliminant une dysparathyroidie. La fonction rénale et la protidémie étaient normales ainsi que le reste du bilan biologique notamment son bilan lipidique. L’ECG a montré une légère hypertrophie ventriculaire gauche et un allongement de l’espace QT. L’évolution à court terme a été marquée par la survenue d’un état de mal convulsif posant l’indication d’une intubation avec sédation continue. L’électroencéphalogramme avait montré un ralentissement de l’activité fond avec la présence de crises convulsives infra cliniques incitant de mettre le patient plus tard sous Lévétiracétam. Le diagnostic retenu était celui de Syndrome de Fahr dû au traitement antiépileptique donnant lieu à une hypocalcémie chronique source de calcification au niveau des noyaux gris centraux.

Le patient a bénéficié d’un traitement à base de sels de calcium (2g par jour) associé à la vitamine D (1500 UI par jour), d'anti coagulation et d'antiagrégant plaquettaire, ce qui a permis un sevrage progressif et extubation au 5e jour. La correction des troubles du métabolisme phosphocalcique a amené à une disparition des crises épileptiques, confirmée notamment par la normalisation de l’EEG de contrôle. Cependant il a gardé sa paralysie faciale ainsi que l’hémiparésie droite. Par la suite, le patient fut transféré au service de neurologie pour suites de soins.

## Discussion

Le syndrome de Fahr est une entité anatomo-clinique rare, caractérisée par la présence de calcifications intracérébrales, bilatérales et symétriques, localisées au niveau des noyaux gris centraux survenant préférentiellement chez les patients présentant des dysparathyroïdies [2]. Peu d’études de prévalence et de morbi-mortalité ont été réalisées sur ce sujet dans le monde et particulièrement en Afrique. Deux difficultés sont rencontrées. La première tient à la proportion inconnue de porteurs asymptomatiques. La seconde tient à la prévalence des calcifications des NGC liées à l'âge en population générale [3].

Le syndrome de Fahr est généralement difficile à suspecter cliniquement car les manifestations cliniques ne correspondent à aucun tableau spécifique. Ce syndrome peut demeurer asymptomatique et être découvert fortuitement. Des troubles neuropsychiatriques sont fréquemment observés, tels que des troubles caractériels et/ou du comportement. D’autres manifestations neurologiques sont possibles mais moins habituelles, comme des troubles cognitifs, une débilité mentale, un syndrome extrapyramidal, des crises convulsives partielles ou généralisées. Plus rarement un syndrome cérébelleux ou pyramidal, un syndrome d’hypertension intracrânienne, une atteinte des nerfs crâniens, une chorée ou des accidents vasculaires cérébraux. Un cas d’AVCH révélant un syndrome de Fahr a été décrit récemment [4] mais à notre connaissance aucun cas d’AVCI permanent n’a été décrit. Dans une étude, Farhat et al. ont rapporté l’observation d’un patient qui a plusieurs accidents vasculaires cérébraux ischémiques transitoires, qui n’a pas d’antécédents du diabète, ni d’hypertension artérielle et sans hypercholestérolémie, et chez qui l’examen clinique a trouvé des mouvements choréoathetosiques [5].

La physiopathologie expliquant la survenue d’AVCI au cours du syndrome de Fahr demeure inconnue. Elle peut être expliquée par les dépôts de calcium extracellulaire au niveau des parois des capillaires et des petits vaisseaux entrainant une diminution de la perfusion tissulaire et une ischémie cérébrale [5]. L’hypoparathyroidie est la cause la plus classique du syndrome de Fahr. Dans notre cas, un taux normal de PTH a permis d’éliminer cette éventualité. D’autres affections peuvent engendrer des calcifications intracérébrales, notamment, les endocrinopathies, les maladies de système, la maladie céliaque, certaines infections (toxoplasmose, rubéole…), les tumeurs cérébrales primitives ou secondaires calcifiées. Au cours de ces différentes pathologies, les calcifications intracérébrales ne sont pas bilatérales et symétriques [6, 7]. L’hypervitaminose D, certaines intoxications (oxycarbonnée et au plomb) et la radiothérapie associée ou non à une chimiothérapie intra-thécale par le méthotrexate ont été mis en cause dans certains cas. Le dosage de la vitamine D ainsi que le contexte clinique du patient n’était pas en faveur de ces derniers.

Les antiépileptiques peuvent interférer avec le métabolisme phosphocalcique [8]. Ils peuvent induire un état de carence en vitamine D en augmentant sa fraction inactive. Ils stimulent le système oxydatif enzymatique microsomial hépatique et augmentent la clairance de la vitamine D et de ses métabolites. La phénytoïne altère également l'absorption intestinale du calcium. Ils pourraient par ce biais générer des calcifications des noyaux gris centraux. Notre patient était sous acide valproïque. L'hypocalcémie observée peut être expliqué par la prise des antiépileptiques, ce qui a favorisé les calcifications au niveau des noyaux gris centraux.

## Conclusion

Un traitement antiépileptique au long cours peut interférer avec le métabolisme phosphocalcique et favoriser les calcifications au niveau des noyaux gris centraux et ainsi induire le syndrome de Fahr qui reste de bon pronostic. La correction des troubles du métabolisme phosphocalcique amène souvent une amélioration notable.

## Conflits d’intérêts

Les auteurs ne déclarent aucun conflit d'intérêt.
